# Efficacy of Calcineurin Inhibition in Children With Steroid-Resistant Nephrotic Syndrome

**DOI:** 10.1016/j.ekir.2025.07.037

**Published:** 2025-07-30

**Authors:** Agnes Trautmann, Jonas Hofstetter, Beata Lipska-Ziętkiewicz, Alexey Tsygin, Iwona Ogarek, Bassam Saeed, Maria Szczepanska, Marta Azocar, Francesco Emma, Fatih Ozaltin, Salim Caliskan, Monica Bodria, Dusan Paripovic, Marcin Tkaczyk, Jun Oh, Mounia Boutaba, Helena Jardim, Alev Yilmaz, Dagmar Csaicsich, Bruno Ranchin, Augustina Jankauskiene, Andrea Pasini, Martin Bitzan, Nazym Nigmatullina, Kalman Tory, Jakub Zieg, Roberta Camilla, Nakysa Hooman, Franz Schaefer, PodoNet Consortium, Mounia Boutaba, Dagmar Csaicsich, Sergey Baiko, Marta Azocar, Monica Galanti, Lina Maria Serna Higuita, Jakub Zieg, Bruno Ranchin, Michel Fischbach, Tinatin Davitaia, Jutta Gellermann, Jun Oh, Anette Melk, Agnes Trautmann, Franz Schaefer, Hagen Staude, Nikoleta Printza, Kalman Tory, Alaleh Gheissari, Nakysa Hooman, Giuseppe Remuzzi, Andrea Pasini, Gian Marco Ghiggeri, Giovanni Montini, Luisa Murer, Francesco Emma, Roberta Camilla, Nazym Nigmatullina, Marina Khvan, Nagima Mustapayeva, Valerie Said Conti, Bilal Aoun, Chebl Mourani, Pauline Abou-Jaoudé, Augustina Jankauskiene, Rimante Cerkauskiene, Anna Wasilewska, Lydia Hyla Klekot, Aleksandra Zurowska, Dorota Drozdz, Iwona Ogarek, Marcin Tkaczyk, Małgorzata Stańczyk, Halina Borzecka, Magdalena Silska, Tomasz Urasinski, Agnieszka Firszt-Adamczyk, Mieczyslaw Litwin, Hanna Szymanik-Grzelak, Anna Medynska, Maria Szczepanska, Alberto Caldas Afonso, Helena Jardim, Adrian Lungu, Larisa Prikhodina, Alexey Tsygin, Dusan Paripovic, Radovan Bogdanovic, Jose Eugenio Cabrero Sevilla, Rafael T. Krmar, Sybille Tschumi, Bassam Saeed, Ali Anarat, Esra Baskin, Nilgun Cakar, Ozlem Erdogan, Z. Birsin Özcakar, Fatih Ozaltin, Onur Sakallioglu, Oguz Soylemezoglu, Sema Akman, Ayse Balat, Faysal Gok, Alev Yilmaz, Salim Caliskan, Cengiz Candan, Sevgi Mir, Ipek Akil, Pelin Ertan, Ozan Özkaya, Mukaddes Kalyoncu, Svitlana Fomina, Roman Sobko, Martin Bitzan, Haneen Saeed Hasan Yamin, Eva Simkova, Loai Akram Eid

**Affiliations:** 1 Division of Pediatric Nephrology, University Center for Pediatrics and Adolescent Medicine, Heidelberg, Germany; 2 Rare Diseases Centre and Clinical Genetics Unit, Department of Biology and Medical Genetics, Medical University of Gdansk, Gdansk, Poland; 3 Department of Pediatric Nephrology, National Medical and Research Center for Children’s Health, Moscow, Russia; 4 Department of Pediatric Nephrology, Jagiellonian University Medical College, Krakow, Poland; 5 Farah Association for Child with Kidney Disease, Damascus, Syria; 6 Department of Pediatrics, School of Medicine with the Division of Dentistry, Zabrze, Poland; 7 Department of Pediatric Nephrology, Hospital Luis Calvo Mackenna-Facultad de Medicina Universidad de Chile, Santiago de Chile, Chile; 8 Department of Pediatric Subspecialties, Nephrology and Dialysis Unit, Children’s Hospital Bambino Gesù, IRCCS, Rome, Italy; 9 Department of Pediatric Nephrology, Nephrogenetics Laboratory, Center for Biobanking and Genomics, Hacettepe University, Ankara, Türkiye; 10 Department of Bioinformatics, Nephrogenetics Laboratory, Center for Biobanking and Genomics, Hacettepe University, Ankara, Türkiye; 11 Pediatric Nephrology Departement, Cerrahpaşa Medical Faculty, Istanbul University-Cerrahpasa, Türkiye; 12 Dipartimento di Medicina Clinica e Sperimentale, University of Studies of Parma, Parma, Italy; 13 Division of Nephrology, Dialysis and Transplantation, IRCCS Giannina Gaslini, Genoa, Italy; 14 Department of Pediatric Nephrology, University Children’s Hospital, Belgrade, Serbia; 15 Department of Pediatrics, Immunology and Nephrology, Polish Mothers Memorial Hospital Research Institute, Lodz, Poland; 16 Department of Pediatric Nephrology, University Children’s Hospital, Hamburg, Germany; 17 Department of Pediatric Nephrology, Nefissa Hammond (ex Parnet) Hospital, Algers, Algeria; 18 Department of Pediatric Nephrology, Centre Hospitalar, Porto, Portugal; 19 Department of Pediatric Nephrology, Istanbul Medical Faculty, Istanbul, Türkiye; 20 Department of Pediatrics, Medical University Vienna, Vienna, Austria; 21 Pediatric Nephrology Unit, Hôpital Femme Mere Enfant, Hospices Civils de Lyon, France; 22 Pediatric Center, Institute of Clinical Medicine, Vilnius University, Lithuania; 23 Pediatric Nephrology and Dialysis Unit, S.Orsola-Malpighi Hospital, Bologna, Italy; 24 Kidney Center of Excellence, Dubai Al Jalila Children’s Hospital, Dubai, United Arab Emirates; 25 Department of Nephrology, Asfendiyarov Kazakh National Medical University, Almaty, Kazakhstan; 26 First Department of Pediatrics, Semmelweis University, Budapest, Hungary; 27 Department of Pediatics, University Hospital Motol, Prague, Czech Republic; 28 Pediatric Nephrology, Dialysis, Transplantation Unit, Regina Margherita Children Hospital, Torino, Italy; 29 Department of Pediatrics, School of Medicine, Aliasghar Clinical Research Development Center, Iran University of Medical Sciences, Tehran, Iran

**Keywords:** calcineurin inhibitor, genetic kidney disease, nephrotic syndrome, steroid resistance

## Abstract

**Introduction:**

We aimed to provide evidence for the efficacy of calcineurin inhibitor (CNI) treatment in children with steroid-resistant nephrotic syndrome (SRNS).

**Methods:**

In 278 SRNS children receiving first-line CNI treatment, cumulative remission and kidney failure incidence were estimated using competing risk analysis. Kaplan-Meier and Cox regression analyses were performed to analyze kidney survival, identify predictors of CNI responsiveness and estimate the cumulative incidence of breakthrough proteinuria episodes on or off CNI treatment. The impact of CNI dosage and trough levels on proteinuria was assessed using multivariable linear-mixed effects modeling.

**Results:**

Within 6 months of CNI administration, proteinuria was reduced by 84% (interquartile range: 80%–87%) in 219 nongenetic SRNS cases and by 58% (42%–70%) in 59 genetic SRNS cases but returned to pretreatment level in the latter group within 9 to 12 months. Whereas complete remission was observed in 91 of 219 nongenetic SRNS cases (42%) and 6 of 59 genetic SRNS cases (10%), remission was sustained in 53 nongenetic (24%) and 2 genetic (3%) cases only. Proteinuria reduction, but not attainment of complete remission, was associated with the use of higher CNI doses. The cumulative risk of breakthrough proteinuria on CNI treatment was 51% (40%–62%) and 65% (54%–75%) after 12 and 24 months, respectively, in nongenetic SRNS. The postdiscontinuation relapse risk in patients with complete remission was 40% (22%–59%) and 50% (30%–69%) after 12 and 24 months, respectively. Kidney survival in nongenetic SRNS was superior in CNI-responsive children (92% vs. 42% at 15 years), independent of breakthrough proteinuria episodes.

**Conclusion:**

Our study provides real-world evidence regarding the extent, dynamics, dose-response relationship, and long-term functional impact of CNI therapy in nongenetic and genetic forms of SRNS.

## Graphical abstract

**Figure:**
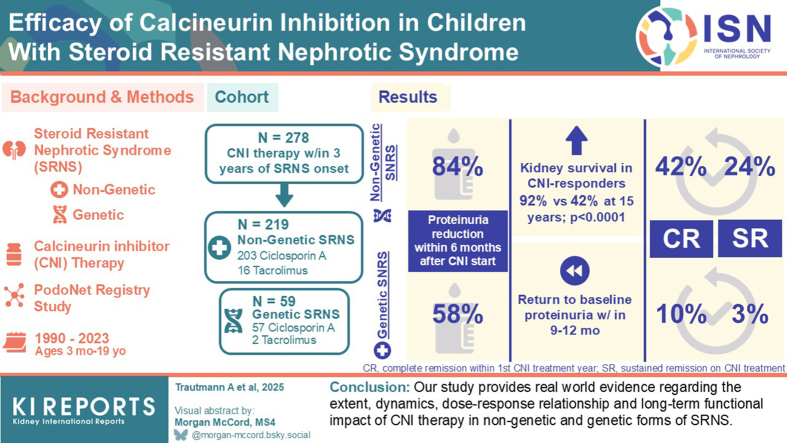

See Commentary on Page 3314

SRNS is a rare and heterogeneous disorder representing 10% to 15% of childhood nephrotic syndrome cases. Twenty percent to 30% of cases are attributable to pathogenic variants in podocyte-associated genes.^1^, ^2^, ^3^, ^4^ The remaining 70% to 80% of “idiopathic” SRNS cases have an unknown etiology. The International Pediatric Nephrology Association (IPNA) Clinical Practice Recommendations advocate for CNI as the primary immunosuppressive therapy.^5^ This recommendation is based on limited evidence from 3 small placebo-controlled randomized clinical trials and 7 randomized clinical trials comparing different immunosuppressive agents in a total of 437 patients.^6^, ^7^, ^8^, ^9^, ^10^, ^11^, ^12^, ^13^, ^14^, ^15^ CNI treatment has shown promising results, with complete and partial response rates ranging from 20% to 77%. Long-term kidney outcomes appear to depend on CNI responsiveness and are poorest in patients with hereditary forms of SRNS.^16^

The role of immunosuppression in hereditary podocytopathies is controversial. The IPNA guideline recommends discontinuing all immunosuppression upon genetic diagnosis confirmation because of general nonresponsiveness and to avoid side effects. However, it has been suggested based on experimental findings and supported by anecdotal clinical observations that CNIs may stabilize the podocyte actin cytoskeleton, potentially leading to partial remission.^17^^,^^18^ Generally, the assessment of the efficacy of CNIs in SRNS has been hampered by the confounding effects of therapeutic renin-angiotensin-aldosterone system (RAAS) blockade.

The PodoNet registry database represents the largest clinical collection of pediatric patients with primary SRNS.^2^ Here, we interrogated the PodoNet registry to provide detailed information, from patients with both nongenetic and genetic disease etiologies, on the extent and temporal dynamics of the antiproteinuric effect of CNI, its persistence both on treatment and after discontinuation, a potential dose-response relationship, and the added antiproteinuric impact of RAAS antagonist therapy. We also addressed the impact of the CNI response type on long-term kidney survival.

## Methods

### Patient Cohort and Analytical Approach

PodoNet is an international registry for childhood-onset primary SRNS, congenital nephrotic syndrome and hereditary podocytopathies. The registry protocol and the description and characterization of the PodoNet cohort has been described elsewhere.^2^ For the current analysis, we selected 278 cohort patients from 19 countries (Supplementary Table S1) aged 3 months to 19 years at disease onset (between 1990 and 2023) with confirmed steroid resistance and documented genetic status who received CNI as first-line SRNS therapy within 3 years of disease onset and had adequately documented proteinuria response information at least during the first treatment year (Supplementary Figure S1). Genetic testing was performed centrally in all patients without a previously confirmed genetic diagnosis and available DNA samples using a custom-made next generation sequencing panel containing 37 podocyte disease-associated genes.

The IPNA SRNS guideline definitions were applied to define complete and partial remission.^5^ Complete remission was defined as proteinuria reduction to < 100 mg/m^2^ 24-hour protein excretion, < 0.2 mg/mg protein-to-creatinine ratio in spot urine (UPCr) (< 0.5 g/g for age < 2 years), a negative dipstick reading, or serum albumin > 30 g/l combined with dipstick trace (+). Partial remission was defined as persistent nonnephrotic-range proteinuria with 24-hour protein excretion > 0.1 but < 1 g/m^2^/d, UPCr of 0.2 to 2 g/g (0.5–2 mg/mg for age < 2 years), dipstick 1+ in combination with serum albumin > 30 g/l or dipstick trace (+) in combination with serum albumin < 30 g/l. Lack of remission was determined as persistent nephrotic-range proteinuria as defined by 24-hour protein excretion ≥ 1 g/m^2^/d, UPCr > 2 g/g, dipstick 2+ or greater as well as dipstick 1+ with serum albumin ≤ 30 g/l. Kidney failure was defined by attainment of chronic kidney disease stage 5 and/or start of renal replacement therapy.

To allow a measure-independent longitudinal description of proteinuria, UPCr equivalent values were derived from 24-hour protein excretion measurements and dipstick measurements. Twenty-four-hour protein excretion values (mg/m^2^/d) were multiplied by 2 to approximate UPCr (g/g). Semiquantitative dipstick measurements were converted to UPCR (g/g) as follows: dipstick 0 and trace: 0.15 g/g, +: 0.5 g/g, ++: 1.5 g/g, and +++: 4 g/g). UPCr values were log-transformed for use in linear mixed-effects models. Sensitivity analyses without UPCr values converted from dipstick measurements yielded the same results.

To allow integrated analyses of associations with ciclosporin A and tacrolimus dose and blood levels, these were categorized into “low,“ “medium,“ and “high“ categories according to the distribution of the time-averaged values (Supplementary Table S2).

### Statistical Analysis

**Figure 1:**
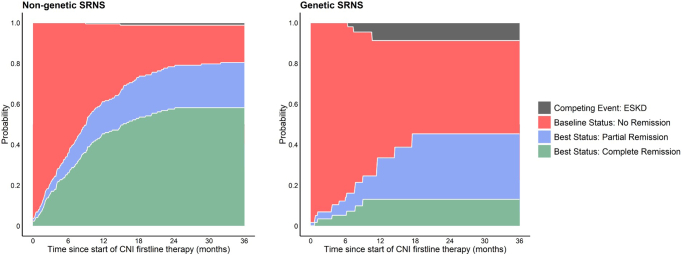
Cumulative incidence of best remission status and kidney failure during CNI treatment (competing risk analysis), stratified by etiology of disease. CNI, calcineurin inhibitor; ESKD, end-stage kidney disease; SRNS, steroid-resistant nephrotic syndrome.

Descriptive statistics are presented as median (interquartile range) for continuous variables and as absolute frequencies and percentages for categorical variables. The evolution of proteinuria within the first CNI treatment year was visualized by applying locally estimated scatterplot smoothing (span: 3 months) on log(UPCr) (Supplementary Figure S2). A competing risk analysis was performed to estimate the cumulative incidence of complete remission, partial remission, and kidney failure while on CNI therapy, stratified by disease etiology (Figure 1). Patients who discontinued CNI treatment without remission were censored at last observation.

**Figure 2:**
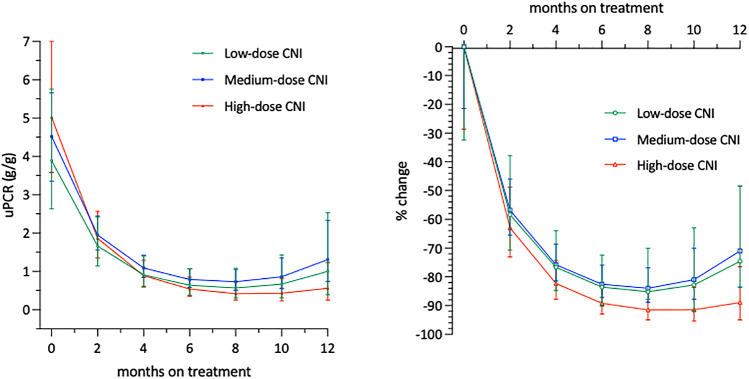
Absolute and relative uPCR change during first year of CNI therapy, stratified by CNI dosage category. CNI, calcineurin inhibitor; uPCR, urinary protein-to-creatinine ratio.

**Figure 3:**
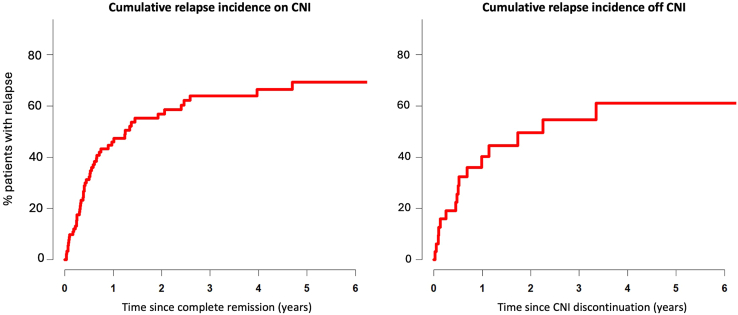
Estimated cumulative incidence of breakthrough proteinuria episodes in patients with nongenetic SRNS who achieved complete remission during first year of CNI therapy. Left panel: Relapse probability while on CNI treatment. Right panel: Relapse probability after CNI discontinuation in patients with sustained complete remission on CNI treatment. CNI, calcineurin inhibitor; SRNS, steroid-resistant nephrotic syndrome.

**Figure 4:**
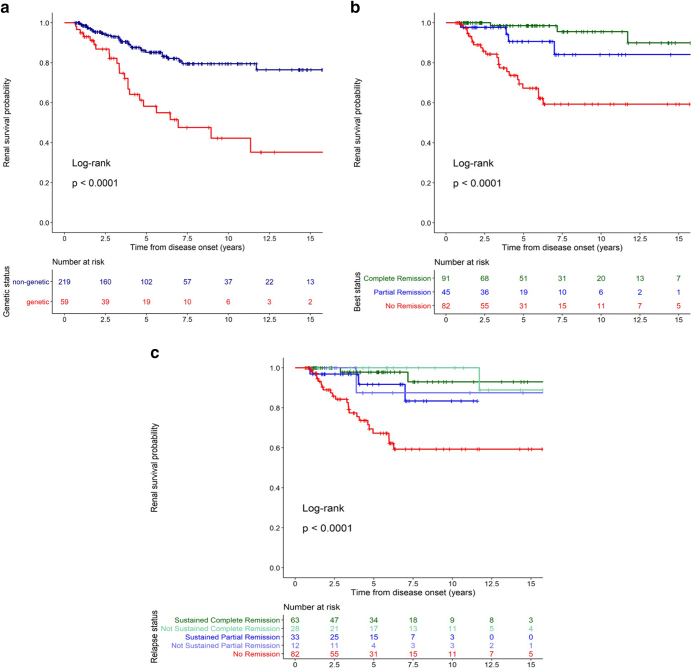
Long-term kidney survival of patients with SRNS treated with CNI. (a) Kidney survival by disease etiology (genetic, red vs. nongenetic, blue). (b) Kidney survival of patients with nongenetic SRNS stratified by best remission status achieved during first treatment year (full remission: green, partial remission: blue, no remission: red). (c) Kidney survival in patients with nongenetic SRNS subgrouped by persistence of remission status (sustained: dark blue green; nonsustained: bright blue/green). CNI, calcineurin inhibitor; SRNS, steroid-resistant nephrotic syndrome.

Factors predicting the attainment of complete remission within the first 12 months of CNI treatment in nongenetic SRNS were evaluated by using univariable and multivariable Cox proportional hazard regression analyses (Supplementary Table S3). Possible associations of CNI dosage and CNI trough levels with proteinuria change was evaluated using multivariable linear mixed-effects models of log(UPCr) (Figure 2, Supplementary Figures S2 and S3, Supplementary Table S4). Linear mixed-effects models were specified with random patient-level intercepts and slopes, and adjusted for baseline patient age and estimated glomerular filtration rate when regressing log(UPCr) on time, time^2^, time-averaged mean or categorized CNI dosage, median or categorized CNI trough levels, and the interaction of time with the respective categorical variable. The cumulative probability of relapsing proteinuria in patients who achieved complete remission within the first CNI treatment year was evaluated by estimating the cumulative incidence of experiencing a first breakthrough proteinuria event since the first observation on complete remission (Figure 3). The long-term kidney functional outcome was evaluated using Kaplan-Meier-analyses (Figure 4). Multivariable Cox regression analysis was used to identify factors predicting kidney survival (Supplementary Table S5). The assumption of proportional hazards was checked for all Cox regression models using formal statistical testing based on the scaled Schoenfeld residuals per model.

## Results

### Patient and Treatment Characteristics

Among 278 SRNS children with first-line CNI immunosuppressive treatment within the first 3 years after disease onset, 59 (21.2%) presented with proven genetic disease (Supplementary Table S6), whereas 219 children had no identified genetic cause. The basic characteristics of the cohort stratified by disease etiology are presented in Table 1. Children with genetic SRNS at disease onset showed a slightly milder initial clinical presentation with less edema and higher serum albumin and a higher proportion of focal segmental glomerulosclerosis than children with nongenetic SRNS. Progression to kidney failure occurred more often in genetic SRNS than in nongenetic SRNS. Information on pharmacological therapies are presented in Table 2. CNI treatment was started on average 2.3 (1.5–5.0) months after disease onset. The vast majority of children were cotreated with oral steroids (96.8 %) and RAAS antagonists (81.3%). In 56 children, mycophenolate mofetil as additional immunosuppressive drug was administered later in the course of disease, less frequently in genetic SRNS (22.8 % vs. 10.2%). Two hundred three of 278 children (92.7%) were treated with ciclosporin A at an average dose of 4.6 (3.6–5.5) mg/kg/d achieving average trough levels of 89 (67–119) ng/ml within the first 12 months of treatment. Sixteen children were started on tacrolimus at an average dose of 0.12 (0.09–0.20) mg/kg/d with mean trough levels of 5.6 (4.3–6.9) ng/ml. CNIs were administered on average for 1.7 (0.8–3.7) years in children with nongenetic SRNS and for 0.7 (0.5–1.2) years in those with genetic disease.

**Table 1:** Patient characteristics

Characteristics at disease onset	Nongenetic	Genetic	Total
	*n* = 219	*n* = 59	*N* = 278
Age groups			
> 3 mo and < 1 yr	14 (6.4%)	7 (11.9%)	21 (7.6%)
≥ 1 and < 6 yrs	122 (55.7%)	33 (55.9%)	155 (55.8%)
≥ 6 and < 12 yrs	50 (22.8%)	10 (16.9%)	60 (21.6%)
≥ 12 yrs	33 (15.1%)	9 (15.3%)	42 (15.1%)
*N*_*i*nfo_	*183*	*49*	*232*
Serum albumin (g/l)	19.5 (15.0–24.0)	25.0 (20.0–30.0)	20.0 (16.0–25.8)
Proteinuria			
*N*_*info*_	*210*	*56*	*266*
Nephrotic range	198 (94.3%)	52 (92.9%)	250 (94.0%)
Nonnephrotic range	12 (5.7%)	4 (7.1%)	16 (6.0%)
Edema			
Severe	63 (28.8%)	10 (16.9%)	73 (26.3%)
Moderate	58 (26.5%)	7 (11.9%)	65 (23.4%)
Mild	42 (19.2%)	14 (23.7%)	56 (20.1%)
None	56 (25.6%)	28 (47.5%)	84 (30.2%)
Kidney function			
*N*_*info*_	*162*	*44*	*206*
eGFR (ml/min per 1.73 m^2^/d)	115 (93–158)	119 (88–169)	115 (91–162)
CKD stage			
CKD 1	127 (78.4%)	32 (72.7%)	159 (77.2%)
CKD 2	26 (16.0%)	5 (11.4%)	31 (15.0%)
CKD 3	8 (4.9%)	5 (11.4%)	13 (6.3%)
CKD 4	1 (0.6%)	2 (4.5%)	3 (1.5%)
Hypertension	48 (21.9%)	16 (27.1%)	64 (23.0%)
Hematuria	97 (44.5%)	27 (45.8%)	124 (44.8%)
Histopathological diagnosis			
*N*_*info*_	*207*	*50*	*257*
FSGS	121 (58.5%)	33 (66.0%)	154 (59.9%)
MCGN	64 (30.9%)	12 (24.0%)	76 (29.6%)
MesPGN	18 (8.7%)	4 (8.0%)	22 (8.6%)
DMS	1 (0.5%)	0	1 (0.4%)
Membranous GN	3 (1.4%)	0	3 (1.7%)
Global glomerulosclerosis	0	1 (2.0%)	1 (0.4%)
*N*_*info*_	*176*	*51*	*227*
Familial disease	24 (13.6%)	11 (21.6%)	35 (15.4%)
Treatment at disease onset			
*N*_*i*__*nfo*_	*174*	*47*	*221*
Daily prednisone duration (mo)	1.3 (1.0–1.7)	1.3 (1.0–1.4)	1.3 (1.0–1.7)
Daily prednisone dose (mg/m^2^/d)	68.7 (46.4–86.1)	74.8 (58.5–89.9)	69.8 (47.8–86.5)
Alternate-day prednisone duration (mo)	1.7 (0.6–4.3)	1.4 (0.8–4.3)	1.6 (0.7–4.3)
Alternate-day prednisone dose (mg/m^2^/48 h)	47.8 (26.1–62.8)	57.4 (37.3–74.8)	50.3 (28.1–65.3)
Follow-up information			
Duration of follow-up (yrs)	4.7 (2.3–7.6)	3.6 (1.7–6.7)	4.3 (2.2–7.5)
Number of patients with kidney failure	32 (14.6%)	24 (40.7%)	56 (20.1%)

CKD, chronic kidney disease; CNI, calcineurin inhibitors; DMS, diffuse mesangial sclerosis; eGFR, estimated glomerular filtration rate; FSGS, focal segmental glomerulosclerosis; GN, glomerulonephritis; MCGN, minimal-change glomerulonephritis; MesPGN, mesangioproliferative glomerulonephritis.

Data are presented as *N* (%) or median (interquartile range).

**Table 2:** Treatment characteristics and response to first-line CNI treatment

Characteristics	Nongenetic	Genetic	Total
	*n* = 219	*n* = 59	*N* = 278
Characteristics at CNI treatment start			
Age (yrs)	4.7 (2.6–10.1)	4.7 (2.4–8.4)	4.7 (2.6–9.8)
Time from disease onset to CNI start (months)	2.1 (1.5–4.2)	3.0 (1.8–8.7)	2.3 (1.5–5.0)
*N*_*info*_	*150*	*39*	*189*
Serum albumin (g/l)	26.0 (20.0–31.2)	21.0 (15.5–27.0)	25.0 (20.0–31.0)
*N*_*info*_	*137*	*37*	*174*
eGFR (ml/min per1.73 m^2^/d)	123 (94–159)	102 (87–159)	118 (91–159)
CNI treatment details			
Duration of CNI treatment (yrs)	1.7 (0.8–3.7)	0.7 (0.5–1.2)	1.4 (0.7–3.2)
Type of CNI treatment			
Ciclosporin A	203 (92.7%)	57 (96.6%)	260 (93.5%)
Tacrolimus	16 (7.3%)	2 (3.4%)	18 (6.5%)
Change of CNI treatment (CsA/tacrolimus)	21 (9.6%)	0	21 (7.6%)
Average CNI drug dosage (mg/kg/d)			
*N*_*info*_	*205*	*56*	*261*
CsA dose	4.62 (3.61–5.46)	4.70 (3.70–6.08)	4.67 (3.63–5.57)
*N*_*info*_	*35*	*2*	*37*
Tacrolimus dose	0.12 (0.09–0.20)	0.10 (0.09–0.10)	0.11 (0.09–0.19)
CNI drug dosage groups			
Ciclosporin A			
*N*_*Info*_	*205*	*56*	*261*
Low dose (< 3.5 mg/kg/d)	43 (21.0%)	10 (17.9%)	53 (20.3%)
Medium dose (≥ 3.5 and ≤ 5.5 mg/kg/d)	113 (55.1%)	26 (46.4%)	139 (53.3%)
High dose (> 5.5 mg/kg/d)	49 (23.9%)	20 (35.7%)	69 (26.4%)
Tacrolimus			
*N*_*Info*_	*35*	*2*	*37*
Low dose (< 0.08 mg/kg/d)	6 (17.1%)	-	6 (16.2%)
Medium dose (≥ 0.08 and ≤ 0.14 mg/kg/d)	15 (42.9%)	2 (100%)	17 (45.9%)
High dose (> 0.14 mg/kg/d)	14 (40.0%)	-	14 (37.8%)
Average CNI trough level			
*N*_*info*_	*145*	*44*	*276*
Ciclosporin A (ng/ml)	89.0 (69.0; 115.2)	91.2 (58.6; 130.7)	89.0 (67.0; 118.8)
*N*_*info*_	*23*	*1*	*24*
Tacrolimus	5.6 (4.3–6.9)	6.3 (-)	5.6 (4.4–6.7)
CNI trough level groups			
Ciclosporin A			
Low level (< 70 ng/ml)	38 (26.2%)	13 (29.5%)	51 (27.0%)
Medium level (≥ 70 and ≤ 100 ng/ml)	68 (46.9%)	13 (29.5%)	81 (42.9%)
High level (> 100 ng/ml)	39 (26.9%)	18 (40.9%)	57 (30.2%)
Tacrolimus			
Low level (< 4ng/ml)	4 (17.4%)	-	4 (16.7%)
Medium level (≥ 4 and ≤ 6 ng/ml)	9 (39.1%)	-	9 (37.5%)
High level (> 6 ng/ml)	10 (43.5%)	1 (100%)	11 (45.8%)
Response to CNI treatment			
12-month proteinuria response			
Complete remission	91 (41.6%)	6 (10.2%)	97 (34.9%)
Partial remission	45 (20.5%)	8 (13.6%)	53 (19.1%)
No remission	83 (37.9%)	45 (76.3%)	128 (46.0%)
Time to best remission (mo)	4.5 (1.8–7.6)	5.4 (1.2–7.6)	4.5 (1.8–7.6)
Any-time proteinuria response			
Complete remission	120 (54.8%)	6 (10.2%)	126 (45.3%)
Partial remission	43 (19.6%)	11 (18.6%)	54 (19.4%)
No remission	56 (25.6%)	42 (71.2%)	98 (35.3%)
Time to complete/partial remission (mo)	6.3 (2.6–11.7)	6.1 (3.6–11.3)	6.3 (2.7–11.6)
Duration of complete/partial remission (mo)	7.0 (3.7–14.2)	5.4 (2.9–8.1)	6.6 (3.6–14.2)
Persistence of remission			
Sustained complete remission	53 (24.2%)	2 (3.4%)	55 (19.8%)
Sustained partial remission	24 (11.0%)	4 (7.6%)	28 (10.1%)
Number of breakthrough proteinuria episodes			
0	77 (47.2%)	6 (35.3%)	83 (46.1%)
1	51 (31.3%)	9 (52.9%)	60 (33.3%)
2	14 (8.6%)	2 (11.8%)	16 (8.9%)
≥ 3	21 (12.9%)	-	21 (11.7%)
Cotreatment with RAAS			
Number of patients (%)	174 (79.5%)	52 (88.1%)	226 (81.3%)
Start of RAAS cotreatment			
Before/at CNI start	115 (66.1%)	36 (69.2%)	151 (66.8%)
After CNI start	59 (33.9%)	16 (30.8%)	75 (33.2%)
Time from disease onset to RAAS initiation (mo)	2.5 (1.1–6.8)	3.0 (1.4–6.6)	2.5 (1.1–6.8)
Type of initial RAAS cotreatment			
ACEi	161 (92.5%)	48 (92.3%)	209 (92.5%)
ARB	7 (4.0%)	2 (3.8%)	9 (4.0%)
ACEi + ARB	6 (3.4%)	2 (3.8%)	8 (3.5%)
Dual RAAS blockade started during course of treatment	42 (19.2%)	12 (20.3%)	54 (19.4%)
Duration of RAAS cotreatment			
> 75%–100% of CNI treatment time	133 (76.4%)	41 (78.8%)	174 (77.0%)
> 50%–75% of CNI treatment time	14 (8.0%)	3 (5.8%)	17 (7.5%)
> 0%–50% of CNI treatment time	27 (15.5%)	8 (15.4%)	35 (15.5%)
Cotreatment with other immunosuppressive agents			
Oral prednisone	213 (97.3%)	56 (94.9%)	269 (96.8%)
MMF	50 (22.8%)	6 (10.2 %)	56 (20.1%)
Steroid pulses	38 (17.3%)	6 (10.2%)	47 (15.8%)

ACEi, angiotensin-converting enzyme inhibitor; ARB, angiotensin 2 receptor blocker; CNI, calcineurin inhibitor; CsA, ciclosporin A; eGFR, estimated glomerular filtration rate; MMF, mycophenolate mofetil; RAAS, renin-angiotensin-aldosterone system.

Data are given as *N* (%) or median (interquartile range).

### Response to CNI treatment

#### Nongenetic SRNS

**Figure 5:**
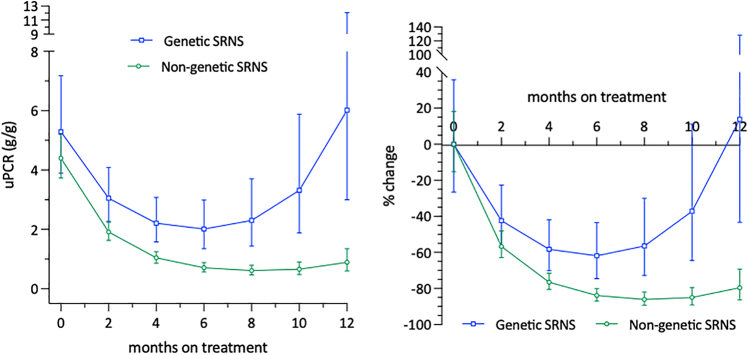
Absolute and relative uPCR change during first year of CNI therapy, stratified by SRNS etiology. CNI, calcineurin inhibitor; SRNS, steroid-resistant nephrotic syndrome; uPCR, urinary protein-to-creatinine ratio.

In the nongenetic SRNS cohort, proteinuria steadily decreased during the first 6 months of CNI therapy, reaching a nadir at 84% (80%–87%) reduction from baseline after 6 months (Figure 5, Supplementary Figure S2). One hundred twenty of 219 children (54.8%) achieved **complete remission** on CNI treatment, 91 within the first treatment year, most likely to be attributed to the CNI effect. The cumulative incidence of complete remission was 45% (37%–53%) at 12 months of CNI treatment, increasing to 57% (50%–64%) within the second year of CNI treatment (Figure 1). Median time to complete remission among the 91 children who remitted in the first treatment year was 4.5 (1.8–7.6) months. In 20 children, complete remission was observed only in the second treatment year, and in 9 children even later, after a median treatment time of 6.3 (4.1–6.7) years. However, all patients in the latter group were on RAAS comedication and the complete remission status was usually transient.

Complete remission was sustained in 53 of 120 children (44%) while on CNI (Table 2). The other 67 children experienced 1 or several episodes of breakthrough proteinuria while on continued CNI treatment. Breakthrough proteinuria occurred after a median of 7.0 (3.7–14.2) months. Forty-three patients (64%) developed nephrotic-range proteinuria and 24 (36%) nonnephrotic-range proteinuria. Fifty of the 67 breakthrough-relapsers (75%) regained complete remission on continued CNI treatment. Among the 91 children who achieved complete remission within the first treatment year, the estimated cumulative incidence of breakthrough proteinuria was 51% (40%–62%) within 1 year after achieving remission, 65% (54%–75%) within 2 years, 71% (61%–82%) within 3 years and reached a plateau at 80% (70%–90%) after 4 years (Figure 3). In 63 of the 120 children who achieved complete remission at any time on CNI, treatment was discontinued. Only 13 of these children (21%) maintained remission, whereas proteinuria recurred in 50 children and immunosuppression was resumed either by restarting CNI and/or second-line immunosuppressive agents. When only the 35 patients with sustained remission achieved on CNI therapy were considered in a time-to-event analysis, the cumulative relapse risk after CNI discontinuation was 40% (22%–59%) at 1 year and increased to 61% (39%–83%) 4 years after CNI discontinuation (Figure 3). The relapsing patients were slightly younger than the nonrelapsers (7.7 [6.1–9.4] vs. 10.6 [7.4–16.2] years).

Forty-three of 219 children (19.6%) achieved **partial remission** on CNI treatment, with a slower reduction of proteinuria than observed in the complete responders (Supplementary Figure S2). In 19 of these patients (44%), nephrotic-range proteinuria reoccurred while on continued CNI treatment.

The 56 of 219 (25.6%) patients who did not even reach partial remission status did not show any relevant change in proteinuria within the first year (Supplementary Figure S2).

In a subgroup of patients with negative genetic testing but familial disease occurrence (24/176 [13.6%]), CNI responsiveness was similar as in those with nonfamilial disease: 12 of 24 (50%) achieved complete remission, 7 (29.2%) partial remission, and 5 (20.8%) were CNI-resistant.

#### Genetic SRNS

In the 59 patients with genetic forms of SRNS, proteinuria was reduced on average by approximately 60% within the first 6 months of CNI administration. This effect was transient, and proteinuria returned to the baseline range within 9 to 12 months (Figure 5).

Six patients achieved complete proteinuria normalization within a median treatment time of 5.0 (1.8–7.4) months, corresponding to a 13% (7%–22%) cumulative incidence of complete remission (Figure 1, Supplementary Table S7). Remission was sustained in only 2 of the 6 children, but their follow-up time on CNI was short (3 and 7 months) (Supplementary Table S7). One of the 2 children had a biallelic *COQ6* pathogenic variant and remained in remission during 4 years of follow-up on coenzyme Q10 therapy, while CNI was stopped after 7 months. Eleven children (18.6%) achieved partial remission status on CNI treatment and concomitant RAAS treatment (8/11); in 7 of those nephrotic-range proteinuria recurred on continued CNI treatment (Supplementary Table S8).

### Factors Associated With CNI Responsiveness

In the nongenetic SRNS cohort, the chance to achieve complete remission within 1 year of CNI treatment was inversely related to the age at disease onset (hazard ratio: 0.93; 95% confidence interval [CI]: 0.88–0.98; *P* < 0.005; Supplementary Table S3). Children aged 1 to 6 years were twice as likely to achieve complete remission as adolescents (hazard ratio: 2.14; 95% CI: 1.06–4.31; *P* < 0.05). By contrast, neither the severity of initial disease manifestation (as evidenced by proteinuria and serum albumin) nor estimated glomerular filtration rate or histopathological findings were predictive of treatment responsiveness.

The likelihood of complete remission was independent of the type and dose of CNI administered and the blood levels achieved but inversely related to the proportion of time of RAAS coadministration (hazard ratio: 0.99; 95% CI: 0.98–0.99; *P* < 0.005). Patients receiving RAAS for 75% to 100% of the CNI treatment time were half as likely to achieve remission as patients without concomitant RAAS treatment (hazard ratio: 0.48; 95% CI: 0.29–0.77; *P* < 0.005), probably reflecting the intensified treatment efforts in patients with lacking responsiveness to CNI treatment.

**Figure 6:**
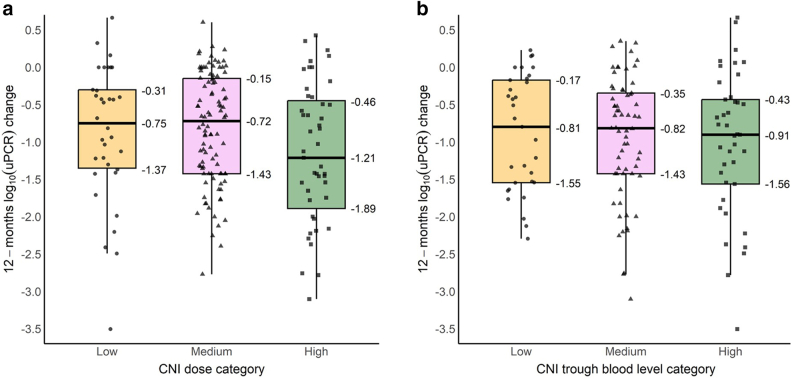
Estimated patient-level log uPCR change during initial 12 months of CNI treatment grouped according to CNI exposure. Colors group patients according to low (orange), medium (purple), or high (green) mean daily dose levels (a) or median trough blood levels (b). Methodological remark: Associations of CNI dosage levels and CNI trough blood level categories with proteinuria reduction in the first year of CNI treatment were evaluated by fitting multivariable linear-mixed effects models with random patient-level intercepts and slopes to patient log_10_(uPCR) values adjusted for baseline age and eGFR. CNI, calcineurin inhibitor; eGFR, estimated glomerular filtration rate; uPCR, urinary protein-to-creatinine ratio.

Whereas complete remission was not affected by the CNI dosing characteristics, absolute and relative proteinuria reduction was greater in the patients with the highest prescribed CNI doses (Figure 2, Figure 6a, Supplementary Figure S3A). This effect increased with time and was confirmed by multivariable analysis to be independent of age and estimated glomerular filtration rate (Supplementary Table S4A). By contrast, CNI trough levels were not associated with proteinuria reduction (Figure 6b, Supplementary Table S4B).

### Long-Term Kidney Outcomes

According to Kaplan-Meier analysis, the proportion of children with nongenetic SRNS with preserved renal function was 85% (95% CI: 80%–91%) at 5 years, 80% (73%–87%) at 10 years and 77% (68%–86 %) at 15 years (Figure 4). The diagnosis of a genetic disease markedly increased the risk of developing kidney failure, with kidney survival rates of 58% (45%–76%) at 5 years, 42% (28%–64%) at 10 years and 35% (20%–61%) at 15 years.

Patients with nongenetic SRNS who attained complete remission on CNI treatment exhibited an excellent long-term outcome with 97% (92%–100%) 10-year kidney survival as compared with 42% (28%–63%) in CNI-resistant patients (*P* < 0.0001) (Figure 4). Children achieving partial remission displayed an intermediate outcome with 77% (59%–100%) 10-year kidney survival. The persistence of complete remission had only a minor impact on long-term kidney survival (Figure 4, Supplementary Table S5). These findings were confirmed by multivariable Cox regression analysis (Supplementary Table S5). Patient age and kidney function at disease onset, the time to achieve remission, the duration of remission, the number of relapses on treatment, and the duration of RAAS cotreatment did not appear to affect long-term kidney survival. The histopathological diagnosis of focal segmental glomerulosclerosis was associated with a higher risk of kidney failure by univariate Cox regression (*P* < 0.05), which was no longer significant when accounting for CNI responsiveness in the multivariate analysis (Supplementary Table S5 and Supplementary Figure S4)

## Discussion

We analyzed this large international pediatric SRNS cohort to provide valid real-world information on the antiproteinuric effect of CNIs, its persistence both on treatment and after discontinuation, a potential dose-response relationship, the added antiproteinuric impact of RAAS antagonist therapy, and the impact of the CNI response on long-term outcome, categorized by the disease etiology.

A substantial global reduction of proteinuria was observed during the first year of CNI therapy. Proteinuria-lowering was more pronounced and sustained in patients with **nongenetic SRNS** than in patients with a genetic disease etiology. A total of 42% of patients with nongenetic disease achieved complete remission during the first year of treatment, confirming previously reported response rates.^2^^,^^16^^,^^19^, ^20^, ^21^, ^22^, ^23^, ^24^, ^25^, ^26^, ^27^, ^28^, ^29^, ^30^, ^31^, ^32^, ^33^, ^34^, ^35^, ^36^, ^37^, ^38^ Proteinuria-lowering mostly occurred within the first 3 to 6 months of CNI therapy, in keeping with previous observations in randomized clinical trials^6^^,^^7^^,^^10^ and observational studies.^21^^,^^26^^,^^35^, ^36^, ^37^, ^38^ However, in > 20% of the patients who achieved complete remission, this only occurred after > 12 months of CNI exposure. Late normalization of proteinuria beyond the first treatment year has also been observed in 15% to 20% of patients after up to 40 months of CNI exposure in a study by Ehrich *et al.*^26^ It appears questionable whether such late disease remissions are causally related to long-term CNI administration. Notably, in a recent analysis of patients with nongenetic SRNS selected from the PodoNet cohort who were never exposed to any immunosuppressants other than initial steroid therapy, a spontaneous remission rate of 58% was observed 2 years after disease onset.^39^

In addition, remission was sustained only in a minority of patients, with up to 80% exhibiting breakthrough proteinuria episodes while on CNI treatment and 80% developing recurrent proteinuria after CNI withdrawal. The observed incidence and timing of breakthrough proteinuria corresponds with reported relapse rates between 31% and 63%^23^^,^^26^^,^^27^^,^^38^ within 4 months to 1 year after achieving complete remission.

To the best of our knowledge, our study is the first to explore CNI exposure-effect relationships in children with SRNS. Whereas a slightly greater quantitative proteinuria reduction was observed in the high-dose category than in patients exposed to low or medium drug doses, no associations with CNI blood levels were found. In addition, the likelihood of achieving complete remission was neither associated with the administered dose nor with the achieved blood levels.

Whereas a significant decline of proteinuria was also observed within 3 to 4 months of CNI treatment in patients with **genetic forms of SRNS**, the reduction was less marked than in the patients with nongenetic SRNS and was not sustained, with proteinuria returning to baseline levels within 9 to 12 months. Complete remission was observed in only 6 out of 59 patients and was short-lasting in all but 1 patient with *COQ6* deficiency who concomitantly received coenzyme Q10 therapy, a highly efficacious treatment of the underlying condition.^40^ Our findings are in keeping with previous cohort studies that typically showed a modest proteinuria-lowering effect of CNI in patients with genetic forms of SRNS, with complete remission observed only in anecdotal cases.^16^^,^^20^^,^^21^^,^^41^, ^42^, ^43^, ^44^ Altogether, our findings support the IPNA clinical practice recommendation to stop immunosuppression when a genetic SRNS cause is identified.^5^

The transient reduction of proteinuria in the genetic SRNS cases may be explained by nonimmunological effects of CNI on proteinuria. Experimental studies have shown a stabilizing effect of CNI on the podocyte actin cytoskeleton.^17^^,^^18^ It is conceivable that such effects may vanish with time as the underlying podocytopathy progresses.

A major confounder to consider in the interpretation of the apparent proteinuria-lowering effect of CNI in SRNS is concomitant **RAAS inhibitor therapy**, which is often coinitiated with CNIs upon diagnosis of SRNS and *per se* may induce a 40% to 50% proteinuria reduction due to a glomerular hemodynamic effect. Concomitant RAAS blockade may explain much of the proteinuria-lowering observed in the patients with genetic SRNS and may have contributed to the results observed in the nongenetic cohort. In the present analysis, it was not possible to disentwine the relative contributions of CNI and RAAS inhibitor therapy to proteinuria reduction due to their almost universal co-administration. Moreover, our findings suggest major bias by indication, with patients with persistent proteinuria being more likely to receive extended RAAS blockade. The recent IPNA clinical practice guideline for SRNS recommends starting RAASi and CNI sequentially to allow distinguishing the effects of the 2 drug classes on proteinuria reduction.^5^

With regard to the **long-term** **outcome** of nongenetic SRNS, apparent CNI sensitivity within the first year of CNI treatment was a clear predictive factor for a favorable renal survival. Similar results were previously reported by the PodoNet cohort^16^ and Gipson *et al.*^22^ for SRNS responsive to intensified immunosuppression within the first year after disease onset. Notably, the favorable predictive effect of achieving complete or partial remission was not compromised by the occurrence of breakthrough proteinuria episodes. The survival benefit associated with proteinuria reduction tended to be present in the genetic SRNS subgroup, although significance was not reached (Supplementary Figure S5). Malakasioti *et al.*^44^ observed a significantly better renal survival in the 25% of a genetic SRNS cohort who achieved at least partial remission within 6 months of CNI treatment. However, the causal relationship remains unclear given the potential confounding by RAAS cotreatment, the timing of diagnosis in the disease course, and the variability of clinical phenotypes.

In addition to the statistical limitations mentioned above, several shortcomings may have impacted this study. These include possible selection bias from voluntary participation, methodological variation in proteinuria reporting, and the variable frequency of longitudinal data entries. Further, because the knowledge about rare genetic causes of SRNS evolved during the observation period and updated comprehensive genetic testing was not possible in all cases, some of the patients might have been incorrectly classified as having a nongenetic disease etiology. Notwithstanding these limitations, we believe that the large size of the international cohort, extensive long-term data collection, and the use of advanced statistical methodologies enabled us to provide information for future reference and reach some meaningful conclusions regarding the role of CNI in pediatric SRNS.

In summary, though the results of our study support the antiproteinuric and nephroprotective efficacy of first-line CNI therapy at least in children with nongenetic forms of SRNS, they also highlight potential pitfalls in the interpretation of CNI effects related to polypragmatic management, potential nonimmunological drug effects, and the potential of spontaneous disease remission. Thus, our findings provide a rationale for non-CNI control groups in future clinical trials and support the use of sequential treatment protocols in clinical practice as suggested in the IPNA SRNS guideline.

## Disclosure

All the authors declared no competing interests.

## Appendix

### List of PodoNet Consortium

Algeria: Mounia Boutaba, Algiers; Austria: Dagmar Csaicsich, Vienna; Belarus: Sergey Baiko, Minsk; Chile: Marta Azocar, Santiago; Monica Galanti, Santiago; Colombia: Lina Maria Serna Higuita, Medellín; Czech Republic: Jakub Zieg, Prague; France: Bruno Ranchin, Lyon; Michel Fischbach, Strasbourg; Georgia: Tinatin Davitaia, Tbilisi; Germany: Jutta Gellermann, Berlin; Jun Oh, Hamburg; Anette Melk, Hannover; Agnes Trautmann, Franz Schaefer, Heidelberg; Hagen Staude, Rostock; Greece: Nikoleta Printza, Thessaloniki; Hungary: Kalman Tory, Budapest; Iran: Alaleh Gheissari, Isfahan; Nakysa Hooman, Teheran; Italy: Giuseppe Remuzzi, Bergamo; Andrea Pasini, Bologna; Gian Marco Ghiggeri, Genova; Giovanni Montini, Milano; Luisa Murer, Padova; Francesco Emma, Rome; Roberta Camilla, Torino; Kazakhstan: Nazym Nigmatullina, Astana/ Almaty; Marina Khvan, Astana, Nagima Mustapayeva, Almaty. Malta: Valerie Said Conti, Malta; Lebanon: Bilal Aoun, Beirut; Chebl Mourani, Beirut; Pauline Abou-Jaoudé, Byblos; Lithuania: Augustina Jankauskiene, Rimante Cerkauskiene, Vilnius; Poland: Anna Wasilewska, Bialystok; Lydia Hyla Klekot, Chorzow; Aleksandra Zurowska, Gdansk; Dorota Drozdz, Krakow; Iwona Ogarek, Krakow; Marcin Tkaczyk, Małgorzata Stańczyk, Lodz; Halina Borzecka, Lublin; Magdalena Silska, Poznan; Tomasz Urasinski, Szczecin; Agnieszka Firszt-Adamczyk, Torun; Mieczyslaw Litwin, Warsaw; Hanna Szymanik-Grzelak, Warsaw; Anna Medynska, Wroclaw; Maria Szczepanska, Zabrze; Portugal: Alberto Caldas Afonso, Porto; Helena Jardim, Porto; Romania: Adrian Lungu, Bucharest, Russia: Larisa Prikhodina, Moscow; Alexey Tsygin, Moscow; Serbia: Dusan Paripovic; Radovan Bogdanovic, Belgrade; Spain: Jose Eugenio Cabrero Sevilla, Cartagena; Sweden: Rafael T. Krmar, Stockholm; Switzerland: Sybille Tschumi, Bern; Syria: Bassam Saeed, Damascus; Türkiye: Ali Anarat, Adana; Esra Baskin, Ankara; Nilgun Cakar, Ankara; Ozlem Erdogan, Ankara; Z. Birsin Özcakar, Ankara; Fatih Ozaltin, Ankara; Onur Sakallioglu, Ankara; Oguz Soylemezoglu, Ankara; Sema Akman, Antalya; Ayse Balat, Gaziantep; Faysal Gok, Gulhane; Alev Yilmaz, Istanbul; Salim Caliskan, Istanbul; Cengiz Candan, Istanbul; Sevgi Mir, Izmir; Ipek Akil, Manisa; Pelin Ertan, Manisa; Ozan Özkaya, Samsun; Mukaddes Kalyoncu, Trabzon; Ukraine: Svitlana Fomina, Kyiv; Roman Sobko, Lviv. United Arab Emirates: Martin Bitzan, Dubai; Haneen Saeed Hasan Yamin, Dubai. Eva Simkova, Dubai, Loai Akram Eid, Dubai.

## Supplementary Material

Supplementary File (PDF) **Figure S1.** Cohort selection from the PodoNet Registry. **Figure S2.** Proteinuria reduction after start of CNI treatment in children with nongenetic SRNS, stratified by best remission status within the first treatment year. **Figure S3.** Proteinuria trajectories according to CNI exposure. **Figure S4.** Kidney survival according to underlying histopathology in patients with nongenetic SRNS. **Table S1.** Number of included patients per country. **Table S2.** Categorization of CNI dosage and trough blood levels. **Table S3.** Factors associated with attainment of complete remission within first CNI treatment year in patients with nongenetic SRNS treated. **Table S4.** Association of CNI dosage and CNI trough levels with proteinuria reduction in the first 12 months of CNI treatment. **Table S5.** Factors predicting kidney survival in nongenetic SRNS. **Table S6.** Distribution of genetic diagnoses in 59 children with hereditary SRNS and proteinuria status modification during CNI therapy. **Table S7.** Characteristics of patients with genetic podocytopathies and reported transient complete proteinuria normalization on CNI therapy. **Table S8.** Characteristics of 11 patients with genetic podocytopathy with partial proteinuria remission on CNI therapy. STROBE checklist.
