# Supplementary material for: Efficacy of Calcineurin Inhibition in Children With Steroid-Resistant Nephrotic Syndrome
Source: Kidney Int Rep. 2025 Jul 30;10(10):3535–48. doi: 10.1016/j.ekir.2025.07.037 (PMC12546756; doi:10.1016/j.ekir.2025.07.037)
Supplement: Supplementary File (PDF) — Figure S1. Cohort selection from the PodoNet Registry. Figure S2. Proteinuria reduction after start of CNI treatment in children with nongenetic SRNS, stratified by best remission status within the first treatment year. Figure S3. Proteinuria trajectories according to CNI exposure. Figure S4. Kidney survival according to underlying histopathology in patients with nongenetic SRNS. Table S1. Number of included patients per country. Table S2. Categorization of CNI dosage and trough blood levels. Table S3. Factors associated with attainment of complete remission within first CNI treatment year in patients with nongenetic SRNS treated. Table S4. Association of CNI dosage and CNI trough levels with proteinuria reduction in the first 12 months of CNI treatment. Table S5. Factors predicting kidney survival in nongenetic SRNS. Table S6. Distribution of genetic diagnoses in 59 children with hereditary SRNS and proteinuria status modification during CNI therapy. Table S7. Characteristics of patients with genetic podocytopathies and reported transient complete proteinuria normalization on CNI therapy. Table S8. Characteristics of 11 patients with genetic podocytopathy with partial proteinuria remission on CNI therapy. STROBE checklist. [file mmc1.pdf]

Efficacy of Calcineurin Inhibition in Children with Steroid Resistant Nephrotic Syndrome

Agnes Trautmann[1], Jonas Hofstetter[1], Beata Lipska-Ziętkiewicz[2], Alexey Tsygin[3], Iwona Ogarek[4], Bassam Saeed[5], Maria Szczepanska[6], Marta Azocar[7], Francesco Emma[8], Fatih Ozaltin[9], Salim Caliskan[10], Monica Bodria[11], Dusan Paripovic[12], Marcin Tkaczyk[13], Jun Oh[14], Mounia Boutaba[15], Helena Jardim[16], Alev Yilmaz[17], Dagmar Csaicsich[18], Bruno Ranchin[19], Augustina Jankauskiene[20], Andrea Pasini[21], Martin Bitzan[22], Nazym Nigmatullina[23], Kalman Tory[24], Jakub Zieg[25], Roberta Camilla[26], Nakysa Hooman[27], Franz Schaefer[1] for the PodoNet Consortium*.

[1] Division of Pediatric Nephrology, University Center for Pediatrics and Adolescent Medicine, Heidelberg, Germany
[2] Rare Diseases Centre and Clinical Genetics Unit, Department of Biology and Medical Genetics, Medical University of Gdansk, Gdansk, Poland
[3] National Medical and Research Center for Children's Health, Moscow, Russia
[4] Department of Pediatric Nephrology, Jagiellonian University Medical College, Krakow, Poland
[5] Farah Association for Child with Kidney Disease, Damascus, Syria
[6] Department of Pediatrics, School of Medicine with the Division of Dentistry, Zabrze, Poland
[7] Pediatric Nephrology, Hospital Luis Calvo Mackenna-Facultad de Medicina Universidad de Chile, Santiago de Chile
[8] Department of Pediatric Subspecialties, Nephrology and Dialysis Unit, Children's Hospital Bambino Gesù, IRCCS, Rome, Italy
[9] Department of Pediatric Nephrology, Department of Bioinformatics, Nephrogenetics Laboratory, Center for Biobanking and Genomics, Hacettepe University, Ankara, Türkiye
[10] Pediatric Nephrology Departement, Cerrahpaşa Medical Faculty, Istanbul University-Cerrahpasa, Türkiye
[11] Dipartimento di Medicina Clinica e Sperimentale, University of Studies of Parma, Parma, Italy and Division of Nephrology, Dialysis and Transplantation, IRCCS Giannina Gaslini, Genoa, Italy
[12] Department of Pediatric Nephrology, University Children's Hospital, Belgrade, Serbia
[13] Department of Pediatrics, Immunology and Nephrology, Polish Mothers Memorial Hospital Research Institute, Lodz, Poland
[14] Department of Pediatric Nephrology, University Children's Hospital, Hamburg, Germany
[15] Department of Pediatric Nephrology, Nefissa Hammond (ex Parnet) Hospital, Algers, Algeria
[16] Department of Pediatric Nephrology, Centre Hospitalar, Porto, Portugal
[17] Department of Pediatric Nephrology, Istanbul Medical Faculty, Istanbul, Türkiye
[18] Department of Pediatrics, Medical University Vienna, Vienna, Austria
[19] Pediatric Nephrology Unit, Hôpital Femme Mere Enfant, Hospices Civils de Lyon, France
[20] Pediatric Center, Institute of Clinical Medicine, Vilnius University, Lithuania
[21] Pediatric Nephrology and Dialysis Unit, S.Orsola-Malpighi Hospital, Bologna, Italy
[22] Kidney Center of Excellence, Dubai Al Jalila Children´s Hospital, Dubai. UAE
[23] Asfendiyarov Kazakh National Medical University, Department of Nephrology, Almaty
[24] 1st Department of Pediatrics, Semmelweis University, Budapest, Hungary
[25] Department of Pediatics, University Hospital Motol, Prague, Czech Republic
[26] Pediatric Nephrology, Dialysis, Transplantation, Regina Margherita Children Hospital, Torino, Italy
[27] Aliasghar Clinical Research Development Center, Department of Pediatrics, School of Medicine, Iran University of Medical Sciences, Tehran, Iran

* see Appendix for collaborators

Running title: SRNS in children

Correspondence address:
Dr. Franz Schaefer
Division of Pediatric Nephrology, Center for Pediatrics and Adolescent Medicine
University of Heidelberg
Im Neuenheimer Feld 430, 69120 Heidelberg, Germany
Email: franz.schaefer@med.uni-heidelberg.de

SUPPLEMENTARY MATERIAL

SUPPLEMENTARY TABLES

Table S-1: Number of included patients per country within this analysis

Country	***No. of patients per country***	***% of patients per country***
Germany	*57*	*20.5*
Poland	*57*	*20.5*
Turkey	*31*	*11.2*
Russia	*29*	*10.4*
Italy	*28*	*10.1*
Syria	*17*	*6.1*
Chile	*15*	*5.4*
Serbia	*14*	*5.0*
Algier	*5*	*1.8*
Portugal	*5*	*1.8*
Austria	*4*	*1.4*
France	*3*	*1.1*
Lithuania	*3*	*1.1*
Czech Republic	*2*	*0.7*
Greece	*2*	*0.7*
Hungary	*2*	*0.7*
UAE	*2*	*0.7*
Iran	*1*	*0.4*
Ukraine	*1*	*0.4*
Total	***278***	***100***

Table S-2: Categorization of Calcineurin inhibitor (CNI) dosage and trough blood levels.

CNI dosages and trough blood levels were grouped into low, medium and high categories based on the distribution of the time-averaged mean values.

	**Ciclosporin A**	**Tacrolimus**
Dosage		
Low	< 3.5 mg/kg/d	< 0.08 mg/kg/d
Medium	≥ 3.5 and ≤ 5.5 mg/kg/d	≥ 0.08 and ≤ 0.14 mg/kg/d
High	> 5.5 mg/kg/d	> 0.14 mg/kg/d
Trough level		
Low	< 70 ng/ml	< 4ng/ml
Medium	≥ 70 and ≤ 100 ng/ml	≥ 4 and ≤ 6 ng/ml
High	>100 ng/ml	>6 ng/ml

Table S-3: Factors associated with attainment of complete remission within first CNI treatment year in patients with non-genetic SRNS treated.

	Univariable analysis			Multivariable analysis		
	HR	***CI_{95%}***	***p***	***HR***	***CI_{95%}***	***p***
Characteristics at disease onset						
**Age** (years)	**0.93**	**0.88-0.98**	**0.004***	0.96	0.90-1.03	0.23
**Age at disease onset** [*ref.* ≥ 12 years]						
>3 months and <1 year	1.69	0.57-5.07	0.34			
**≥1 year and < 6 years**	2.14	1.06-4.31	0.033*			
≥ 6 years and < 12 years	1.32	0.58-3.01	0.52			
**eGFR** (ml/min/1.73m^2)	0.99	0.99-1.001	0.09			
**Serum albumin** (g/l)	0.97	0.94-1.001	0.06			
**Nephrotic-range proteinuria**	0.75	0.31-1.86	0.54			
**Histopathology** [*ref. MCGN*]						
FSGS	0.72	0.45-1.14	0.16	0.69	0.40-1.19	0.18
Other	0.58	0.26-1.33	0.20	0.57	0.23-1.43	0.23
Treatment characteristics						
**Use of Tacrolimus** *[ref. CsA]*	0.91	0.40-2.08	0.82			
**CNI dose category** *[ref. medium]*						
Low	0.95	0.54-1.68	0.87	1.52	0.75-3.07	0.24
High	1.31	0.82-2.09	0.25	1.40	0.76-2.56	0.28
**CNI trough level category** [*ref. medium*]						
Low	0.91	0.51-1.62	0.75	0.67	0.35-1.27	0.22
High	0.69	0.38-1.24	0.21	0.85	0.45-1.58	0.60
**% time on RAAS co-treatment**	**0.99**	**0.987-0.996**	**0.0008***	**0.99**	**0.984-0.996**	**0.0018***

Table S-4: Association of CNI dosage and CNI trough levels with proteinuria reduction in the first 12 months of CNI treatment.

Table S-4A: Multivariable linear mixed-effects model of log uPCR for 180 children with available mean CNI dose levels.

Covariate	$\hat{\boldsymbol{\beta}}$	$\boldsymbol{CI_{95\%}}$	***p***
(Intercept)	0.65	0.55, 0.76	< .0001
Age at baseline (years)	0.001	-0.017, 0.018	.95
eGFR at baseline	0.000	-0.002, 0.001	.60
Time on treatment (years)	-2.46	-2.85, -2.06	<.0001
(Time on treatment)^2 (years^2)	1.91	1.52, 2.31	<.0001
Mean CNI dose category			
- Low	-0.06	-0.27, 0.14	0.53
- Medium	---	---	---
- High	0.06	-0.13, 0.24	0.54
Treatment duration* Mean CNI dose category			
- Time*Low	-0.06	-0.53, 0.41	0.80
- Time*High	-0.44	-0.84, -0.03	.036

Table S-4B: Multivariable linear mixed-effects model of log uPCR for 138 children with available median CNI blood trough levels.

Covariate	$\hat{\boldsymbol{\beta}}$	$\boldsymbol{CI_{95\%}}$	***p***
(Intercept)	0.62	0.49, 0.75	< .0001
Age at baseline (years)	-0.003	-0.020, 0.015	.77
eGFR at baseline	0.000	-0.001, 0.001	.87
Treatment duration (years)	-2.65	-3.10, -2.20	<.0001
Treatment duration^2 (years^2)	2.03	1.61, 2.46	<.0001
Median CNI blood level category			
- Low	-0.11	-0.33, 0.11	.32
- Medium	---	---	---
- High	0.19	-0.01, 0.38	.06
Treatment duration * CNI blood level category			
- Time*Low	-0.003	-0.53, 0.53	.99
- Time*High	-0.18	-0.65, 0.30	.46

Methodological Remarks:
The linear mixed-effects models (LMM) were specified with random patient-level intercepts and slopes to account for within-patient correlation. Restricted Maximum Likelihood was used to estimate the LMM´s fixed effect β parameters. The LMM was fitted to data from patients with at least two repeated (converted) uPCR during CNI therapy; patients who already achieved remission at the first reported uPCR value on treatment were excluded. The time effect in the model represents the estimated linear slope in $\log_{10}$(uPCR) of patients in the medium CNI dosage category.

Table S-5: Factors predicting kidney survival in non-genetic SRNS.

In the non-genetic SRNS subgroup, univariable and multivariable cox regression analyses were performed to identify predictive factors for renal survival.

	**Univariable Analysis**			**Multivariable Analysis**		
**Variable**	**HR**	**95% CI**	**p**	**HR**	**95% CI**	**p**
**Characteristics at disease onset**						
Age (years)	1.04	0.96-1.13	0.36	1.01	0.90-1.13	0.86
eGFR (ml/min/1.73m^2)	1.00	0.99-1.01	0.96	1.00	0.99- 1.01	0.95
Histopathology (ref: MCGN)						
FSGS	**2.83**	**1.07-7.45**	**0.04***	1.53	0.47-5.00	0.48
Other	2.69	0.64-11.36	0.18	1.80	0.38-8.52	0.46
Familial disease	0.91	0.27-3.05	0.88			
**12-month proteinuria response**						
(*Ref.: no remission)*						
Complete remission	**0.09**	**0.03-0.30**	**<0.0001***	**0.06**	**0.01-0.45**	**0.0063**
Partial remission	**0.27**	**0.09-0.78**	**0.016***	0.28	0.08-1.01	0.053
Time to best remission (weeks)	0.99	0.95-1.05	0.89			
Duration of best remission **(**weeks)	0.99	0.99-1.00	0.28			
**Persistence of best remission status on CNI**						
(Ref.: no remission)						
Sustained complete remission	**0.09**	**0.02-0.39**	**0.0011***			
Non-sustained complete remission	**0.08**	**0.01-0.62**	**0.015***			
Sustained partial remission	**0.29**	**0.09-0.96**	**0.043***			
Non-sustained partial remission	0.23	0.03–1.70	0.15			

Table S-6: Distribution of genetic diagnoses in 59 children with hereditary SRNS and proteinuria status modification during CNI therapy.

Causative gene	Frequency N (%)	Transient/sustained complete remission	Transient/sustained partial remission
NPHS2	29 (49.2%)	3 /1	3/ 1
WT1	11 (18.6%)	1 / -	6/ 3
SMARCAL1	6 (10.2%)	-	-
LMX1B	3 (5.1%)	-	2/ -
COQ6	2 (3.4%)	1 / 1	
INF2	2 (3.4%)	-	-
COL4A5	1 (1.7%)	-	-
COQ2	1 (1.7%)	1 / -	-
COQ8B (ADCK4)	1 (1.7%)	-	-
MYO1E	1 (1.7%)	-	-
LAMB2	1 (1.7%)	-	-
TRPC6	1 (1.7%)	-	-
**Total**	**59 (100%)**	**6 (10.2%) / 2 (3.4%)**	**11 (18.6%) / 4 (6.8%)**

Table S-7: Characteristics of patients with genetic podocytopathy and reported transient complete proteinuria remission on CNI therapy.

Abbreviations: CNI = Calcineurin-Inhibitors, MCGN = minimal-change glomerulonephritis, FSGS = focal segmental glomerulosclerosis, DMS = Diffuse mesangial sclerosis, RAAS antagonist = renin-angiotensin aldosterone system antagonist, CKD = chronic kidney disease

Gene	Causative variant(s)	Age at disease onset (yrs)	S-albumin at disease onset (g/l)	Histo-pathology	Time from 1st manif. to CNI start (yrs)	Duration of CNI 1st line treatment (yrs)	RAAS co-treatment (% CNI treatment time)	Time to remission (yrs)	Duration of remission (yrs)	Sustained remission on/off CNI	Follow-up (yrs)	Remission status at last observation	Status at last observation
NPHS2	c.[353C>T];[353C>T]	0.6	19	-	1.6	0.6	100	0.3	0.2	Yes/ no	9.3	No rem.	CKD1
NPHS2	c.[868G>A];[868G>A]	7.9	30	MCGN	0.5	0.9	100	0.5	0.3	No/-	1.5	Partial rem.	CKD1
NPHS2	c.[365G>C];[851C>T]	5.4	28	MCGN	0.2	1.0	86	0.6	0.1	No/no	4.8	No rem.	Kidney failure/PD
WT1	c.1447+5G>A	4.3	40	FSGS	1.0	2.2	100	0.1	0.5	No/no	6.7	No rem.	CKD2
COQ6	c.[803delC];[1078C>T]	2.4	38	FSGS	0.1	1.0	74	0.7	3.4	yes/ yes	4.3	Compl. rem.	CKD1
COQ2	c.[571-1G>A];[683A>G]	2.1	22	FSGS	0.2	6.7	100	0.1	3.3	No/no	6.9	Partial rem.	CKD1

The reference transcripts for variant description are: *COQ2* -NM_001358921.2; *COQ6* - NM_182476.3; *NPHS2* - NM_014625.4; *WT1* - NM_024426.6.

Table S-8: Characteristics of 11 patients with genetic podocytopathy with **partial** proteinuria remission **on CNI therapy**.
Abbreviations: see Table S2.

Gene	Causative variant(s)	Age at disease onset (yrs)	S-albumin at disease onset (g/dl)	Histo-pathology	Time from 1st manif. to CNI start (yrs)	Duration of CNI treatment (yrs)	Time to partial remission (yrs)	Duration of partial remission (yrs)	RAAS co-treatment (%CNI treatment time)	Sustained partial remission while on/off CNI	Follow-up (yrs)	Remission status at last observation	Status at last observation
NPHS2	c.[249del];[538G>A]	3.9	27	MesPGN	0.5	2.1	0.1	1.5	44	No/-	2.6	Partial rem.	CKD1
NPHS2	c[467dup];[467dup]	0.9	20	MCGN	0.1	1.0	1.0	-	92	Yes/-	1.2	-	CKD4
NPHS2	c.[686G>A];[890C>T]	13.4	30	MesPGN	1.5	0.9	0.5	0.2	19	No/no	3.3	No rem.	ESRD/ PD
WT1	c.1447+4C>T	1.7	10	FSGS	0.2	1.6	1.2	0.4	-	No/-	1.8	Partial rem.	CKD2
WT1	c.1447+4C>T	8.0	29	FSGS	0.3	0.8	0.3	0.5	-	Yes/no	6.9	No rem.	ESRD/Tx
WT1	c.1447+5G>A	1.0	-	FSGS	2.2	9.9	8.4	2.3	-	Yes/no	17.1	No rem.	CKD5
WT1	c.1366T>C	3.2	32	FSGS	0.1	3.8	0.1	3.6	97	Yes/no	12.0	No rem.	CKD2
WT1	c.1447+2T>C	5.4	31	MCGN	2.6	0.6	0.4	0.2	86	No/no	3.6	Partial rem.	CKD2
WT1	c.1387C>T	15.3	25	FSGS	0.3	2.5	1.0	0.5	39	No/-	2.8	No rem.	CKD3
LMXB1	c.737G>C	9.4	26	FSGS	0.7	6.8	0.6	0.5	95	No/-	7.5	Partial rem.	CKD1
LMXB1	c.737G>C	4.1	26	MCGN	0.7	6.8	1.5	0.7	95	No/-	7.5	Partial rem.	CKD2

The reference transcripts for variant description are: *LMX1B* - NM_001174147.2*; NPHS2* - NM_014625.4; *WT1* - NM_024426.6.

SUPPLEMENTARY FIGURES

Figure S-1: Cohort selection from PodoNet Registry

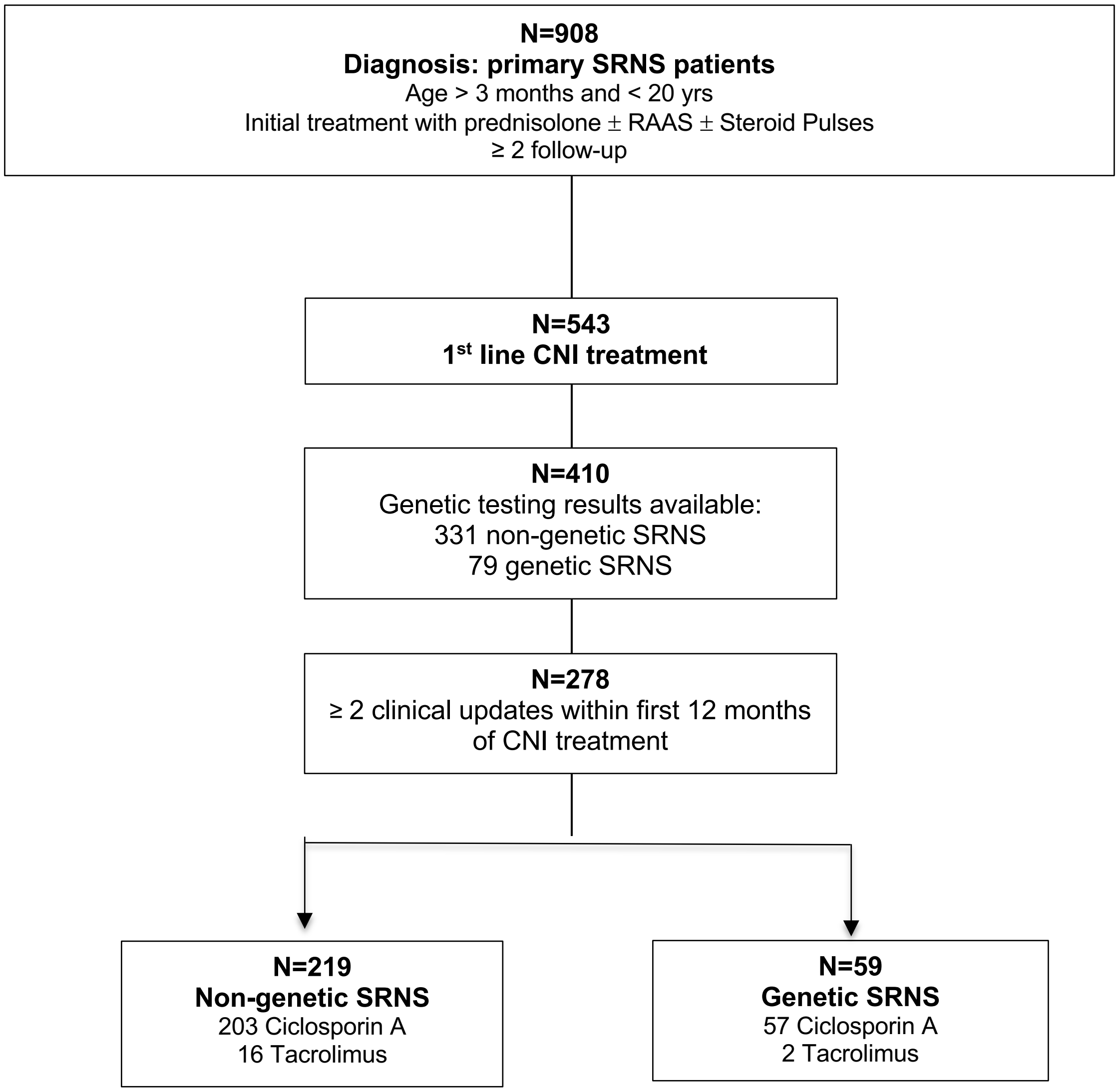

Figure S-2: Proteinuria reduction after start of CNI treatment in non-genetic SRNS children, stratified by best remission status within the first treatment year. Complete remission level is indicated by the interrupted line.

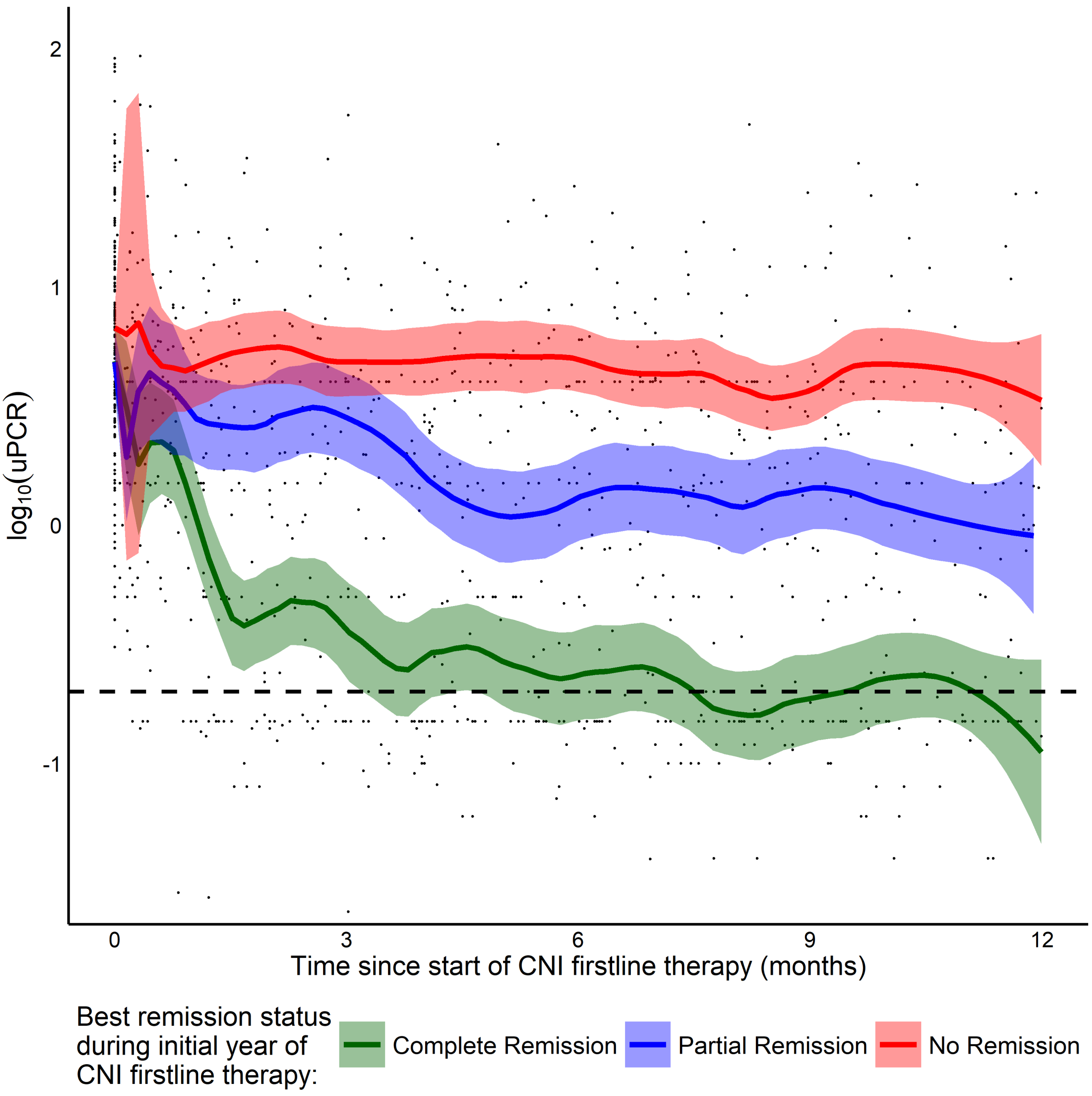

Figure S-3: Proteinuria trajectories according to CNI exposure.
Coloured lines represent average proteinuria courses of patients with low (orange), medium (purple), or high (green) mean daily dose levels (***Panel a***) or median trough blood levels (***Panel b***) as measured during the initial 12 months of CNI therapy. Black lines represent individual patients´ fitted proteinuria trajectories.

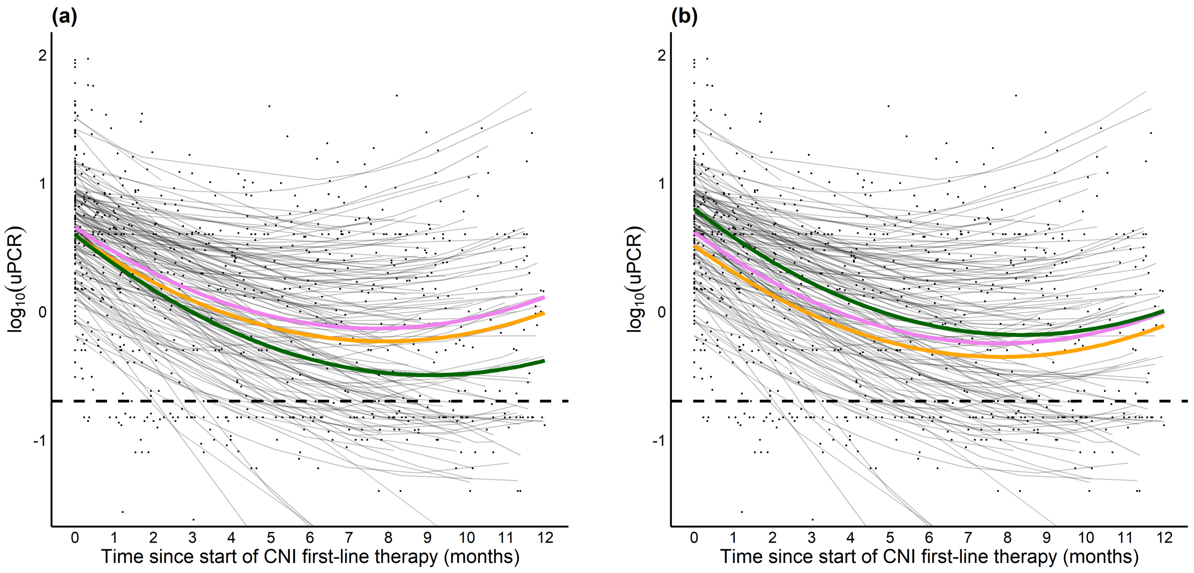

Methodological remark: The association of CNI dosage levels and of CNI trough blood levels (categorized into low, medium, and high dose levels and trough levels, respectively; see Table S4) with proteinuria reduction in the first year of CNI treatment was evaluated by comparing fitted $\log_{10}$(uPCR) average trajectories as modelled by a multivariable linear-mixed effects model, adjusting for baseline age and eGFR with random patient-level intercepts and slopes.

Figure S-4: Kidney survival according to underlying histopathology in patients with non-genetic SRNS.

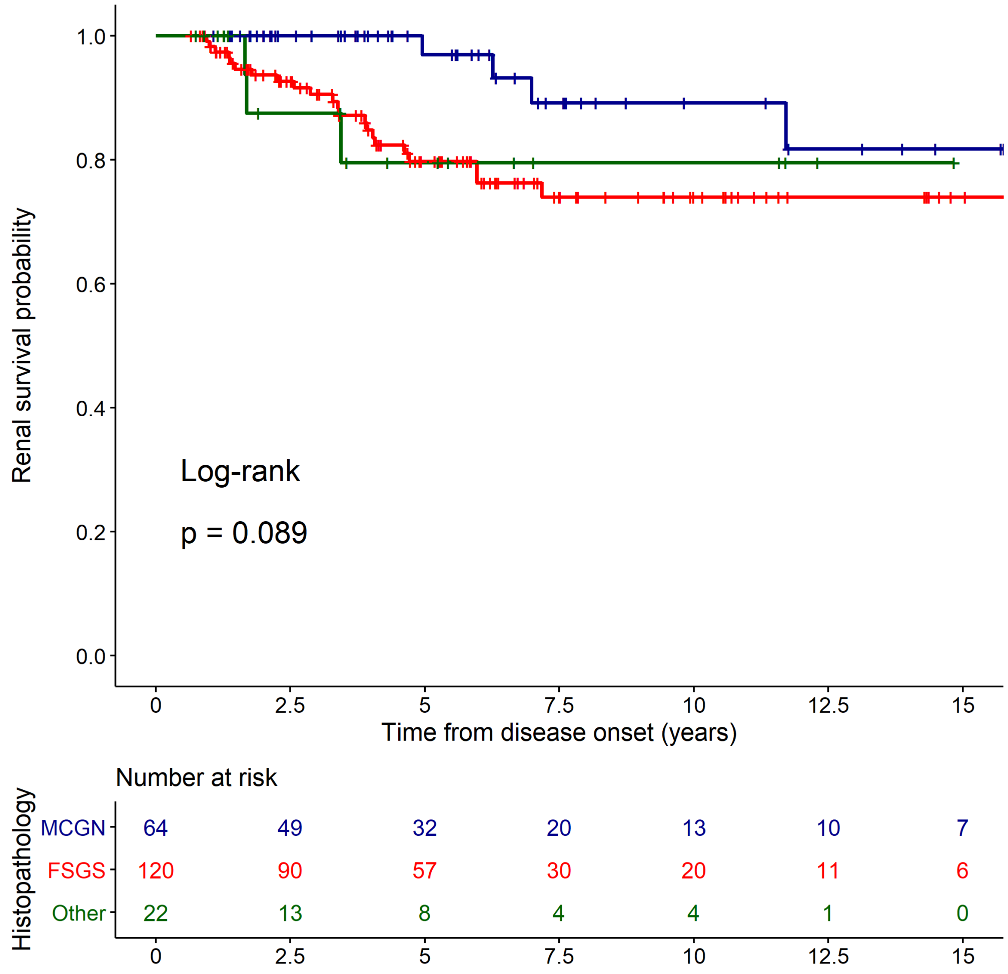

Figure S-5: Kidney survival in genetic SRNS stratified by best remission status achieved within first year of CNI treatment.

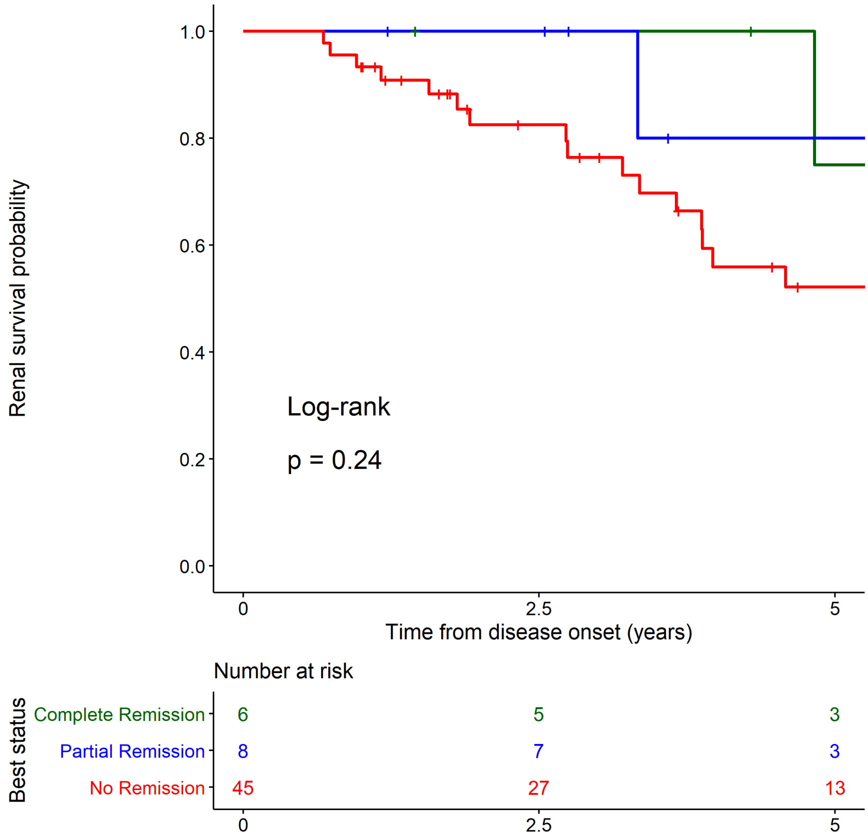

STROBE Statement—Checklist of items that should be included in reports of ***cohort studies***

	Item No	Recommendation	Page No
**Title and abstract**	1	(*a*) Indicate the study's design with a commonly used term in the title or the abstract	1
		(*b*) Provide in the abstract an informative and balanced summary of what was done and what was found	2
**Introduction**			
Background/rationale	2	Explain the scientific background and rationale for the investigation being reported	3
Objectives	3	State specific objectives, including any prespecified hypotheses	3
**Methods**			
Study design	4	Present key elements of study design early in the paper	4
Setting	5	Describe the setting, locations, and relevant dates, including periods of recruitment, exposure, follow-up, and data collection	4
Participants	6	(*a*) Give the eligibility criteria, and the sources and methods of selection of participants. Describe methods of follow-up	4, Fig S-1
		(*b*) For matched studies, give matching criteria and number of exposed and unexposed	
Variables	7	Clearly define all outcomes, exposures, predictors, potential confounders, and effect modifiers. Give diagnostic criteria, if applicable	4
Data sources/ measurement	8*	For each variable of interest, give sources of data and details of methods of assessment (measurement). Describe comparability of assessment methods if there is more than one group	4-5
Bias	9	Describe any efforts to address potential sources of bias	5, 12
Study size	10	Explain how the study size was arrived at	4, Fig S-1
Quantitative variables	11	Explain how quantitative variables were handled in the analyses. If applicable, describe which groupings were chosen and why	4
Statistical methods	12	(*a*) Describe all statistical methods, including those used to control for confounding	5
		(*b*) Describe any methods used to examine subgroups and interactions	
		(*c*) Explain how missing data were addressed	
		(*d*) If applicable, explain how loss to follow-up was addressed	
		(*e*) Describe any sensitivity analyses	
**Results**			
Participants	13*	(a) Report numbers of individuals at each stage of study—eg numbers potentially eligible, examined for eligibility, confirmed eligible, included in the study, completing follow-up, and analysed	Fig S-1
		(b) Give reasons for non-participation at each stage	
		(c) Consider use of a flow diagram	
Descriptive data	14*	(a) Give characteristics of study participants (eg demographic, clinical, social) and information on exposures and potential confounders	Table 1+2
		(b) Indicate number of participants with missing data for each variable of interest	
		(c) Summarise follow-up time (eg, average and total amount)	
Outcome data	15*	Report numbers of outcome events or summary measures over time	9

Main results	16	(*a*) Give unadjusted estimates and, if applicable, confounder-adjusted estimates and their precision (eg, 95% confidence interval). Make clear which confounders were adjusted for and why they were included<br>(*b*) Report category boundaries when continuous variables were categorized<br>(*c*) If relevant, consider translating estimates of relative risk into absolute risk for a meaningful time period	6-9<br>Table 1+2
Other analyses	17	Report other analyses done—eg analyses of subgroups and interactions, and sensitivity analyses	6-9
**Discussion**			
Key results	18	Summarise key results with reference to study objectives	10-12
Limitations	19	Discuss limitations of the study, taking into account sources of potential bias or imprecision. Discuss both direction and magnitude of any potential bias	12
Interpretation	20	Give a cautious overall interpretation of results considering objectives, limitations, multiplicity of analyses, results from similar studies, and other relevant evidence	10-12
Generalisability	21	Discuss the generalisability (external validity) of the study results	12
**Other information**			
Funding	22	Give the source of funding and the role of the funders for the present study and, if applicable, for the original study on which the present article is based	13

*Give information separately for exposed and unexposed groups.

Note: An Explanation and Elaboration article discusses each checklist item and gives methodological background and published examples of transparent reporting. The STROBE checklist is best used in conjunction with this article (freely available on the Web sites of PLoS Medicine at http://www.plosmedicine.org/, Annals of Internal Medicine at http://www.annals.org/, and Epidemiology at http://www.epidem.com/). Information on the STROBE Initiative is available at http://www.strobe-statement.org.
